# Patient feedback on hospital pharmacists’ consultation skills: A feasibility study using the Interpersonal Skills Questionnaire (ISQ)

**DOI:** 10.1371/journal.pone.0268544

**Published:** 2022-07-14

**Authors:** Hiyam Al-Jabr, Michael J. Twigg, Thando Katangwe-Chigamba, Robin Saadvandi, James A. Desborough

**Affiliations:** 1 Integrated Care Academy, University of Suffolk, Ipswich, United Kingdom; 2 School of Pharmacy, University of East Anglia, Norwich, United Kingdom; 3 Norwich Medical School, University of Eact Anglia, Norwich, United Kingdom; 4 Norfolk & Norwich University Hospital, Norwich, United Kingdom; National Taiwan University College of Medicine, TAIWAN

## Abstract

**Background:**

Improvement in practitioners’ consultation skills (CSs) can be driven by patient feedback, however, to date, no study has been conducted with reference to pharmacy consultations. The Interpersonal Skills Questionnaire (ISQ) is potentially appropriate for collecting patient feedback on pharmacists’ CSs. This study aims to explore the feasibility of collecting patient feedback on hospital pharmacists’ CSs using the ISQ, to identify the acceptability of the feedback process, and to identify methods to enhance the process in the future.

**Methods:**

The study was conducted in a teaching hospital, United Kingdom, between 2018 and 2019. A diverse sample of pharmacists with patient-facing roles was purposively selected. The study comprised three phases. Pharmacists collected feedback from patients following their consultation using the ISQ utilising a third person whenever possible (phase-1). Data analysis and individual report writing was conducted by a private company. Interviewing a sample of patient participants by telephone (phase-2), and interviewing pharmacists face-to-face after receiving feedback reports (phase-3). All interviews were transcribed verbatim and thematically analysed. The study received approval by the NHS Health Research Authority.

**Results:**

Six pharmacists were included. Of the 119 distributed ISQs, 111 were returned (response rate 93%). Patients were mostly recruited by their consulting pharmacists (72%, n = 80). All pharmacists and 14 patients were interviewed. Participants were positive about patient feedback and its role in enhancing CSs. Most did not encounter any problem with the process, however, some pharmacists struggled to find a third person. The ISQ was mostly viewed suitable to assessing pharmacy consultations. Some reports highlighted areas to improve (e.g. protecting patient’s privacy).

**Conclusions:**

Collecting feedback is feasible, acceptable and may enhance CSs, however, the process was associated with challenges such as finding a third person. Several measures should be considered to make the process more feasible within the hospital pharmacy setting.

## Background

Patient feedback has been used since the 1980s by different healthcare organisations for the purpose of enhancing the quality of healthcare [1]. In the United Kingdom (UK), enhancing the quality of healthcare is a major focus of the National Health Service (NHS), and since 2002, patient feedback has been increasingly contributing to assessing healthcare in England [2, 3] and has been widely acknowledged for its benefits [4–6]. The 2019 NHS business plan aimed to put patients at the centre of the healthcare system with a view to shaping services around their needs [7].

Providing practitioners with patient feedback with reference to their individual performances can help them in identifying their strengths and weaknesses [8–10] which they can then use to enhance their professional development. A previously conducted systematic review [11] provides evidence that improvements in practitioners’ consultation skills (CSs) can be driven by patient feedback, such as increasing the explanations they give to patients regarding their treatment [12], and increasing quality time spent during consultations [13]. However, in spite of the increased attention towards patient feedback, there is still lack of evidence with using it with pharmacy professionals [11]. With the increasing number of patient-facing roles and consultations conducted by pharmacists in hospitals [14–17], providing them with a tool to collect patient feedback could help them in improving their consultations. Several tools were identified by the systematic review, however, one tool showed more promise for it to be used with pharmacists. This tool had better validity and reliability compared to other tools examined, and its general characteristics made it a suitable candidate to be taken forward (e.g. a simple tool that is easy to understand, takes less than three minutes to complete, and has a free text for patients to write their suggestions). It was designed using different approaches that helped in reflecting what is perceived important from patients’ perspectives in relation to CSs of practitioners. A generic form of the tool, the Interpersonal Skills Questionnaire (ISQ), was pre-tested in a think-aloud study with a group of patients following their consultation with a hospital pharmacist [18]. Findings indicated that the ISQ is potentially suitable to be taken forward and used in assessing pharmacy consultations. Therefore, this study aimed to investigate the feasibility of collecting patient feedback on hospital pharmacists’ CSs. The objectives were to identify: (1) the acceptability of the feedback process to all participants, (2) pharmacists’ subsequent actions after receiving the feedback report, and (3) methods to enhance the practicality of collecting patient feedback in this setting.

## Methods

### Study design and location

This was a single-centre study conducted at a large teaching hospital in the East of England, UK, between July 2018 and March 2019. It received ethical approval by the NHS Health Research Authority (IRAS 240348). A mixed-methods approach was used and the study was conducted in three phases (the first two ran concurrently): collecting patient feedback on pharmacists’ CSs using the ISQ (phase-1), interviewing a sample of patients who participated in phase-1 (phase-2), and interviewing pharmacists and pharmacists’ colleagues (with whom the report was discussed) (phase-3).

### The ISQ questionnaire

The ISQ is a 13 item anonymous questionnaire that assesses CSs of practitioners. It uses a 5-point Likert scale (poor to excellent) and takes less than three minutes to complete. The questionnaire also includes a free text question for patients to write their comments. Completed ISQs were sent to the Client Focused Evaluations Program (CFEP) UK surveys who own the ISQ for data analysis and report writing. CFEP issued validated and abbreviated reports, depending on the number of completed questionnaires returned per pharmacist. An aggregated report was also issued to the research team to provide an overview of all feedback. Benchmarks (average scores from the ISQ from other healthcare professionals, based on data held by the company) were provided in the validated and aggregated reports. Validated and aggregated reports presented mean score percentages for each item of the ISQ (See Table 1 below for calculation of mean score percentages).

**Table 1:** Example of mean score calculation in patient feedback report.

Q1) Satisfaction with visit to the pharmacist (total number of responses to Q1 = 30)						
ISQ rating scale	Poor	Fair	Good	Very good	Excellent	Non rated responses
Number of ratings	0	0	5	9	16	0
Value assigned to each rating	0	25	50	75	100	n/a

[(number of Poor ratings x 0) + (number of Fair ratings x 25) + (number of Good ratings x 50) + (number of Very Good ratings x 75) + (number of Excellent ratings x 100)] ÷ [(total number of patient responses—number of Non-rated responses)] = mean score of Q1. = [(0 x 0) + (0 x 25) + (5 x 50) + (9 x 75) + (16 x 100)] ÷ [(30–0)], thus, mean percentage score for Q1 = 84%.

### Pharmacists and patient participants

As informed by literature [19–22] and within available resources, a 10% sample of hospital pharmacists were included. At that time, there were 59 hospital pharmacists, therefore, six pharmacists were recruited.

Pharmacists who regularly conduct patient consultations were invited to participate by email and those who showed interest were purposively recruited to obtain a diverse sample based on their gender, years of qualification, and clinical area worked in at the hospital. Selected pharmacists attended an information session to discuss recommended methods of feedback collection as derived from literature [11] and to obtain their consent.

Eligible patients were those ≥ 18 years old and present in the hospital for a consultation with the pharmacist (either as an inpatient or outpatient). Patients who could not communicate in English, who were not suitable for inclusion (e.g. have cognitive impairment), or who stayed at the hospital more than four days after the pharmacist’s consultation were excluded.

### Data collection

#### Phase-1

Pharmacists were asked to collect patient feedback (using a third person, e.g. ward nurse, pharmacy technician, where possible) within one hour of the consultation (to reduce patients’ recall bias and feedback contamination by other consultations) and to complete a questionnaire administration form. They were also directed to collect feedback from ≥ 25 patients each to obtain validated feedback [23]. A three month duration was given for this phase.

#### Phase-2

During phase-1, patients were also invited by their pharmacists to phase-2. Up to 18 patients (average three per pharmacist) were targeted to be interviewed (as guided by reaching data saturation for the whole group). Interviews were conducted less than two weeks after the pharmacist’s consultation. Patients received a £10 amazon voucher for their participation.

#### Phase-3

All pharmacists were interviewed in phase-3 and one colleague per pharmacist (with whom the report was discussed) was anticipated to be interviewed to explore their views about patient feedback.

Interview topic guides for phase-2 and 3 (Table 2) were developed in accordance with the study aim, objectives and feasibility focus areas. Interviews were audio-recorded.

**Table 2:** Topic guides at patients’ and pharmacists’ interviews.

Patient interview topic guide	Pharmacist interview topic guide	Pharmacist’s colleague topic guide
1. What do you think about the consultation you have had with the pharmacist you assessed? • What was good/not so good about the pharmacist’s consultation you have assessed? • What do you think of the ISQ as a tool to assess pharmacy CSs? (Relevance to pharmacy CSs)*.	1. Tell me about your thoughts of conducting consultations with patients? Likes / dislikes	1. Tell me what do you think about collecting patient feedback regarding CSs of pharmacists? • Role of patient feedback • How do you think patient feedback tools could be used/integrated in the usual practice of the pharmacist? • How do you think it should be administered?
2. Tell me about your experience with patient feedback**. • Who gave you the ISQ? • How did you return your completed ISQ to the marked box? • Describe any concerns or worries you might have encountered during the process? • What do you think could have been done differently when collecting your feedback?	2. How do you normally get feedback on your consultation? • Feelings about using patient feedback • Views about ISQ as an assessment tool*	2. How did the pharmacist introduce the patient feedback report to you? • How did you learn about the report? In a formal meeting or informal/friendly chat?
3. What would you like to see happening as a result of this feedback?	3. Can you please describe the method(s) you used for questionnaire administration?** • Use of third person • Encountered barriers • Do you think that the method used for questionnaire administration might have influenced patients’ ratings? How? Why do you think so? • - How do you think barriers could be overcome to facilitate a better implementation of the process in the future?	3. What do you think about the value of this report? • To pharmacists undergoing the assessment? • To patients? • To you as a colleague/peer/or line manager? • How do you think the report could be used? • - Do you think this process / feedback report could drive changes to practice? Why? Why not? How?
4. If collecting feedback from patients to pharmacy consultations becomes frequent, will you be encouraged to give your feedback again? Why/why not?	4. Tell me what happened when you received your report. • Ease of reading and understanding • Usefulness to identify strengths and weaknesses • Discuss results with others, who, why? • What changes did you do or plan to do following reading your report? If no changes conducted/planned ask why?	4. How do you think the report could be used if there was a negative feedback? • What kind of support can you provide to the pharmacist based on your role (as a colleague/peer/ or line manager)? (thing you can do to help pharmacist improve areas with negative feedback)
	5. What would you do differently if you are going to use patient feedback again? • Facilitators • Need to discuss results with someone • Whom do you recommend to discuss your report with? Why? When do you think it should take place?	5. What do you think about using the report as part of the pharmacist’s appraisal / or for formal revalidation process?

* Questions on the ISQ tool.

** Questions on the feedback process.

### Data analysis

The ISQs were analysed by the CFEP and feedback reports were generated. Descriptive data analysis was conducted by the researcher for all participants using data collected by feedback reports and the questionnaire administration forms.

Audio-recordings of interviews were transcribed verbatim by the researcher and/or a transcriber assistant. Transcripts were anonymised, coded and thematically analysed to identify common emerging themes [24]. Inductive thematic analysis approach was used. Transcripts were continuously revisited and the accuracy was verified by listening to recordings and comparing it with the transcripts. Coding of data was conducted using NVivo® software. Coded transcripts were checked by another member of the research team (MT and/or TK) to ensure appropriate and consistent coding process. Any disagreements were resolved by consensus, and by referring to the transcripts and original recordings. Final themes were presented to the research team, and were supported by anonymised quotes from the different participants.

### Feasibility areas of focus

Several areas of feasibility were identified [25]. However, the approach to assessing CSs was considered feasible when meeting the following areas: acceptability (i.e. willingness of pharmacists to receive and patients to give feedback and the likely patient response rate), applicability (i.e. identifying applicable method(s) for questionnaire administration), and practicality (i.e. pharmacists’ views of feedback reports and the intention of using it).

## Results

### Phase-1

#### Questionnaire administration

Six pharmacists were included (50% females) with median age (interquartile range (IQR)) of 27 years (25, 31). All pharmacists worked across inpatient and outpatient settings.

Out of 119 distributed ISQs, 111 were returned (response rate = 93%). Most patients were recruited from an inpatient setting (n = 75, 68%). Sixty one (55%) participants were ≥ 60 years old. See Table 3 for more details. More information on data collected is presented in S1 Appendix).

**Table 3:** Details of recruited patient participants (N = 111).

	Total No. (%)
Age*	
Under 25 years	3 (3%)
25–59 years	42 (38%)
Over 60 years	61 (55%)
Blank/spoilt	4 (4%)
First time to have a consultation with the pharmacist*	
Yes	94 (85%)
No	9 (8%)
Blank/spoilt	7 (6%)
Total no.	110 (99%)

* One patient did not report age or whether this is the first time to have a consultation with the pharmacist.

Three pharmacists collected feedback from ≥ 25 patients over a period of 8–11 weeks. Eighty (72%) ISQs were administered by the consulting pharmacists. Five pharmacists used a third person on 31 (28%) occasions, especially when recruiting inpatients (n = 25, 23%). See Table 4 for more details.

**Table 4:** Description of patients approached per each pharmacist.

Pharmacist code	No. patients approached	No. ISQs returned (response rate %)	Inpatients (No., %)	Female gender (No., %)	1^st^ time to have a consultation with this pharmacist (No., %)
Ph-A	36	30 (83%)	30 (100%)	14 (47%)	27 (90%)
Ph-B	10	10 (100%)	10 (100%)	6 (60%)	9 (90%)
Ph-C	9	8 (89%)	2 (25%)	5(63%)	6 (75%)
Ph-D	7	7 (100%)	1 (14%)	4 (57%)	1 (14%)
Ph-E	34	28 (82%)	17 (61%)	15 (54%)	26 (93%)
Ph-F	29	28 (97%)	15 (54%)	16 (57%)	25 (89%)
Total	125	111 (89%)	75 (68%)	60 (54%)	94 (85%)

#### Feedback reports

**Fig 1:**
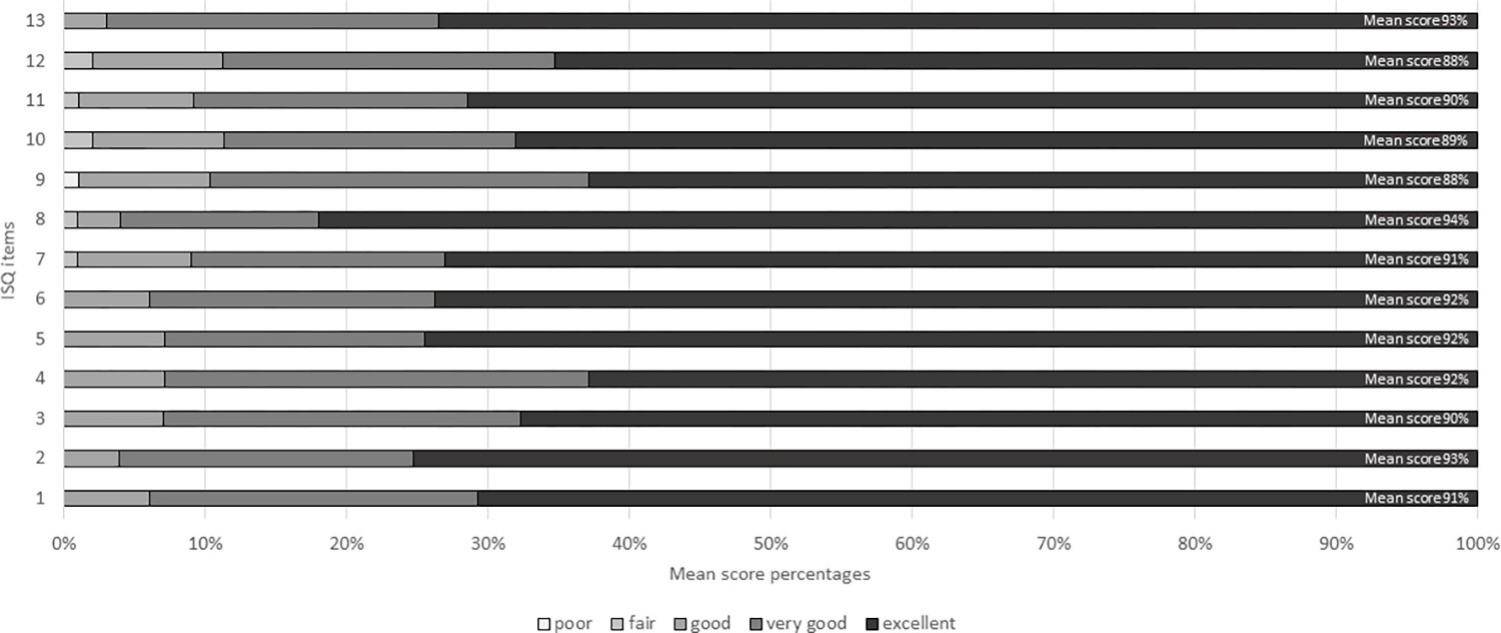
Scores given by patients for each item of the ISQ (N = 111). ISQ items: 1. Satisfaction with visit to the pharmacist, 2. Warmth of the pharmacist’s greeting, 3. The pharmacist’s ability to really listen, 4. The pharmacist’s explanations of things, 5. Extent to which patient felt reassured, 6. Confidence in the pharmacist’s ability, 7. Opportunity given to express concerns/fears, 8. Respect shown by this pharmacist, 9. Amount of time given for this visit, 10. Consideration of personal situation, 11. Pharmacist’s concern for patient as a person, 12. Extent the pharmacist helped patient to self-care, 13. Recommendation patient would give to friends.

Three validated, three abbreviated and one aggregated feedback reports were issued. In the former, mean score percentages ranged from 84% (item 12) to 96% (items six, eight and 13). Written comments were generally positive with only four highlighting areas to consider, such as protecting patients’ privacy. Pharmacists’ mean feedback scores were highly comparable to benchmarks (Fig 1).

### Phase-2

Four pharmacists recruited patients for phase-2 and 14 were interviewed (50% females) (see Table 5). The median (IQ) age of patients was 68 years (58, 77). The majority recruited (71.4%, n = 10) were inpatients. Interviews lasted on average 14 minutes.

**Table 5:** Details of patients participating in phase-2.

Patients’ codes	Age	Gender	Inpatient / outpatient	ISQ administration	ISQ collection
P-1	67	Male	Inpatient	By a 3^rd^ person (another pharmacist)	By a 3^rd^ person
P-2	62	Female	Inpatient	By a 3^rd^ person (another pharmacist)	By a 3^rd^ person (another pharmacist)
P-3	79	Female	Inpatient	By a 3^rd^ person (another pharmacist)	Could not remember
P-4	66	Male	Inpatient	Pharmacist	By same pharmacist
P-5	55	Female	Inpatient	Pharmacy technician	Left it on bed (thus collected by a 3^rd^ person)
P-6	76	Male	Inpatient	Pharmacist	By a 3^rd^ person
P-7	54	Female	Outpatient	Pharmacist	By a 3^rd^ person (receptionist)
P-8	59	Male	Inpatient	Pharmacist	By same pharmacist
P-9	83	Female	Inpatient	Pharmacy technician	By a 3^rd^ person (Another pharmacist)
P-10	81	Male	Inpatient	Pharmacist	By a 3^rd^ person (a nurse)
P-11	43	Male	Outpatient	Pharmacist	By a 3^rd^ person (receptionist)
P-12	69	Male	Outpatient	Pharmacist	By same pharmacist
P-13	71	Female	Inpatient	Pharmacist	By a 3^rd^ person (Left envelope on side table)
P-14	72	Female	Outpatient	Pharmacist	By a 3^rd^ person

Five overarching themes presented through the data, these are described below with supportive quotes.

#### Theme-1: Opinions on pharmacists

All patient participants described their experience with the pharmacist’s consultation as being generally positive, and well delivered. Patient participants commented on how friendly pharmacists were, which helped in making them feel comfortable during the consultation. The majority also described a different set of CSs pharmacists used, which they appreciated, such as providing explanations in a simple language, and dedicating enough time to answer their questions.

*“that’s right ya ya he wasn’t rushed or anything like that he gave…I didn’t feel that he was rushing to get passed you know get me spoken to him and then move on to somebody else or umm ya” (P-5)*.

A minority of patient participants acknowledged the new roles pharmacists are currently undertaking which contributed in making them more visible and approachable to patients than before. A participant stressed the importance for pharmacists to be used as a point of reference on issues regarding medication since they are the medication experts.

#### Theme-2: Views on the feedback process

Most patient participants felt positive about the feedback process without encountering any problem. They also highlighted being able to complete the questionnaire in their own time without being rushed. Most reported receiving the ISQ by their pharmacists at the end of the consultation, with brief explanation about the study and assurance that their participation is voluntary.

*“it was absolutely fine I mean [pharmacist] presented it very well and…explained it very clearly, there was no pressure, she made it very clear” (P-14)*.

Patients reported responding to the ISQ honestly regardless of who gave it to them. Although the majority were supportive of the feedback process, some expressed concerns over some aspects with suggestions to improve it. These included the confidentiality when approaching patients, timing of approaching patients and options to return completed questionnaires. Suggestions were to approach patients privately, if possible approach them earlier during their hospital stay or allow them take the questionnaire and return it by post and to approach patients enough time after a surgery. A final suggestion was to have someone return to collect the completed questionnaire, especially at the point of patient discharge.

#### Theme-3: Comments on the ISQ

Findings indicated that all patient participants viewed the ISQ as a simple questionnaire that is easy to understand and complete. Most agreed on its relevance/suitability for assessing pharmacy consultations. One participant described in depth that the ISQ highlights the skills pharmacists use in their consultations with patients.

*“yes the questionnaire it’s it’s open …. it’s a reflection on know what you think of the care that was given and why you think that care was given, it gives you the opportunity to actually air what you feel about the pharmacist and any problems that you had with the pharmacist and how they behaved towards you, I think it’s very important you know they treated as an individual that’s’ what’s highlighted in the actual questionnaire you know their approach to people this is all good” (P-8)*.

However, only one participant did not view it to be very relevant and that its questions need to be revised.

*“I don’t think it’s very reflective about that to be honest, the questions could be more in depth, could be more relevant, could be more thought out…but it needs to be perhaps a bit more relevant I mean perhaps needs less questions but more in depth more or more pointed more thought out” (P-14)*.

This participant also gave a few suggestions on how to improve the questionnaire including reducing the number of items by combining some items together and also increasing the size of the text box to write more comments. The participant reasoned her views to her profession, where she used to work with and criticise questionnaires.

#### Theme-4: Benefits of patient feedback

A number of benefits were mentioned by patient participants regarding the collected feedback. Reported benefits were related to patients themselves, to pharmacists, and to healthcare services. Patient participants agreed that feedback will bring many benefits, they valued being asked to give feedback and some described it as a way that helps in expressing their feelings.

*“I think it’s very important…very important you know people experience in the hospital clinic outpatients whatever, it’s very important that they get feedback they get a say in uh you know what’s happening in their lives medication wise” (P-8)*.

A minority reported that this process made them satisfied as they contributed to helping other people. Most agreed that feedback could benefit pharmacists by highlighting areas to improve. Feedback also allows pharmacists to know that they are appreciated for their work, which will then keep them motivated to high working standards to maintain this cycle of satisfaction.

The experience of collecting feedback was hoped to motivate other patients to increase the level of trust and confidence in their pharmacists and to rely on them for getting information about drug therapy without feeling that they should always ask their doctors.

#### Theme-5: Willingness and desire to continue to give feedback

This was a distinct theme but relatively short that all patient participants mentioned in a similar way. All patient participants were very supportive and expressed their willingness and agreement to give feedback again in the future. They reasoned that to all the benefits they foresee for the feedback besides their willingness to give help to whoever needs it. Some patient participants also recommended the continuation of this process of collecting feedback, especially that this was a new thing for them to experience.

*“well I’d like to see it continued because from a patient point of view it’s nice to know what’s going on as I said I’ve been in hospital before I never seen anything like this” (P-4)*.

### Phase-3

Seven interviews were conducted, six with pharmacists and one with a pharmacist’s colleague. Five main themes presented through the data, these are described below with supportive quotes.

#### Theme-1: Challenges to conducting patient consultations

Pharmacists perceived consultations as an opportunity to increase patients’ understanding of their own treatment, answer their questions and help in driving positive outcomes such as improving adherence.

*“they can present to you information that they haven’t discussed with the doctor or the nursing staff…and that’s quite nice to find that you can offer them something that might make the difference” (Ph-F)*.

Pharmacists also shared challenges they sometimes encounter such as decreased patients’ understanding about their roles and insufficient skills to interact effectively with difficult patients. Some mentioned receiving little training at university when compared to other practitioners. Given suggestions included introducing more placements to the pharmacy degree and encouraging pharmacists to raise patients’ awareness about their roles.

#### Theme-2: Views on questionnaire and study process

Pharmacists viewed the ISQ as a simple tool and most perceived it relevant to assess pharmacy consultations. However, two pharmacists viewed some items as not being always applicable to all patients, indicating that it may need fine adjustments such as by adding a “not-applicable” answer option.

Several challenges were encountered with the feedback process. These included busy workload and forgetting to take the ISQs to recruitment site. Suggestions included collecting feedback electronically, using reminders to take the questionnaire, or storing it at recruitment sites.

*“it is just me remembering a lot of the time to take the box up to the clinic…and the distance from clinic to pharmacy is…I mean you can’t just pop back and get it not if I even remembered I just got caught up so maybe you know a reminder to take the box to clinic” (Ph-D)*

With respect to study duration, a range between 3–5 months was considered sufficient for collecting feedback from the target number of patients. Additionally, pharmacists suggested to have feedback collected every 1–2 years.

#### Theme-3: Challenges and suggestions for patient recruitment

Using a third person was the biggest challenge which made pharmacists administer most ISQs by themselves. Only one pharmacist reported not using a third person. The difficulty of finding a third person was due to their busy workload and shortness of staff. Additionally, the need to explain the study to each new third person was time consuming.

*“because the nurses are always switching to then go up and every time like explain or I think you’d kind of end up repeating the same thing again and again it would take up so much time” (Ph-E)*

Pharmacists agreed that using a third person would resolve these challenges and make the process more feasible. The third person may also encourage patients to give honest responses and enhance collecting feedback from those with reading/writing difficulties. Increasing the awareness of other team members of the feedback collected could help in providing pharmacists with a third person to assist the process. This could be someone who is based in the area from where feedback is to be collected (e.g., a pharmacy technician, another pharmacist or any other pharmacy team member, a ward clerk or a clinic receptionist), or, where possible, an external person could be specifically assigned to support this. However, this will need to be agreed and arranged within the pharmacy team to identify how to best achieve this.

#### Theme-4: Factors inducing potential response bias

Most pharmacists agreed that recruiting patients themselves might have derived favourable responses. One pharmacist perceived feedback collected at the hospital to be probably not a true reflection of the pharmacist’s performance.

*“there are lots of different things can influence their opinions…it might not be particularly related to the service I gave but they might just be a little bit unhappy with the clinic as a whole” (Ph-D)*

Two pharmacists described using a consistent patient recruitment approach to reduce selection and response bias. The majority reported recruiting patients randomly, sometimes selecting some patients over others. Suggestions included to follow a consistent recruitment approach and use a third person when possible.

#### Theme-5: Report usefulness and subsequent action

Varying views were given about reports’ usefulness. Those who received validated reports identified areas that needed attention and that they have started already in responding to it.

*“I noticed that with time I’d forgotten to ask patients if it was ok to do that now…actually I need to remember to say is it ok if I do that now because they might need the loo or they might be going for a scan or something you just don’t know” (Ph-A)*

Also, provided benchmarks were described to be helpful. Although pharmacy specific benchmarks were preferred, pharmacists acknowledged that such data is not yet available and that this process could help in creating specific ones to use in the future.

A number of barriers were reported by some to hinder the usefulness of reports. These included the lack of negative feedback and the lack of specific comments to justify low scores.

Most pharmacists reported discussing the report with a colleague(s). Discussions helped in clarifying how to improve. Different ways were indicated by the pharmacist’s colleague to support pharmacists like guiding them to useful online resources.

## Discussion

This is the first exploratory study conducted with hospital pharmacists with respect to pharmacy consultations and patient feedback. It adds novel information to this under researched area and provides insights on how to improve the process in the future. Findings support the feasibility of collecting patient feedback on hospital pharmacists’ CSs and on providing pharmacists with feedback reports.

The concept of patient feedback was acceptable to all participants who also acknowledged its benefits in improving performance. This aligns with other studies where practitioners were also in favour on the role patient feedback plays in their development [13, 26–40].

Findings also indicate patients’ agreement to give feedback since 93% of the ISQs were returned. Several factors might have contributed to this, including the characteristics of the ISQ [41] and the use of a face-to-face recruitment approach [42, 43].

For some pharmacists it was feasible to collect feedback from the target number of patients, however, the practicality of the process for all pharmacists was hindered by a number of challenges. Most were related to their busy workload and time schedule, limited availability of third persons and the inconsistent recruitment. Such challenges made pharmacists recruit most patients themselves, with some selecting certain patients over others, thus introducing selection bias.

Results support the feasibility of providing pharmacists with feedback reports, however, views were varied regarding its usefulness. This was attributed to the specificity of feedback and its ability to highlight areas to improve, which aligns with the findings of other studies that these were factors influence responding to feedback and changing one’s practice [44–49].

Pharmacists who found reports to be useful (especially the validated reports), mentioned that the feedback highlighted specific areas to focus on. However, some pharmacists, especially those who received the abbreviated reports described them as being of limited usefulness. This was influenced by the nonspecific positive comments to guide improvement. The positivity of given scores might have created a ‘ceiling effect’, which is usually associated when most scores accumulate towards the favourable end of the response scale [50]. The ceiling effect was described to be associated with nonspecific feedback that makes it difficult for professionals to differentiate or identify areas to focus on [51]. This probably contributed in decreasing the usefulness of these reports as these pharmacists could not identify how or what to do to improve their consultations, since given scores were already in the upper end of the scale thus indicating no further development is needed.

Discussing reports with colleagues was helpful with improving performance. Similar findings were reported elsewhere [49], where discussions helped in clarifying areas needing attention and in designing action plans.

Participants agreed that the ISQ is simple and mostly relevant to assess pharmacy consultations. This confirms the outcome of the think-aloud study [18] and supports its face validity. However, some recommended fine adjustments to become more relevant to hospital pharmacy (e.g., adding a “non-applicable” answer option to the questionnaire).

Suggestions to enhance collecting feedback included those given by patients to approach them at the right time and place and to collect the completed questionnaire from them. Privacy is a challenge in a hospital environment. To help maintain patient’s privacy when collecting feedback, where possible, patients could be moved to a private area to have appropriate discussion and give them enough time to complete the questionnaire privately and confidentially. However, as private areas are not always available, other options could be considered such as allowing patients to take the questionnaire to complete it at their own time and then post it back. Besides maintain privacy, this could also avoids adding extra burdens on patients to wait to return the completed questionnaire, yet, it may increase the risk recall bias and feedback contaminated by consultations conducted by other healthcare professionals. Suggestions to enhance collecting feedback that were given by pharmacists were focused on assigning a third person to help with feedback collection and to collect feedback every 1–2 years. Similar to their counterpart healthcare professionals, providing more training to pharmacists during their undergraduate studies was also mentioned to help them be better equipped with the necessary CSs. This was also suggested to help in promoting people’s awareness about pharmacists and their role in patients’ care.

### Strengths and limitations

The study has several strengths to highlight. It used mixed methods and the included sample of patients encompassed a diversity of characteristics, thus providing wider views on the practicality of conducting the process. Efforts were also made to reduce the effects of feedback contamination by other practitioners’ consultations through approaching patients for feedback within one hour of the consultation. Additionally, the study represents a steppingstone towards building pharmacy specific benchmarks for future use.

However, some limitations were encountered. These include conducting the study in a single hospital with a small number of pharmacists and patients, which may thus limit the generalizability of results. Additionally, selection bias and potentially response bias were introduced by pharmacists when recruiting patients themselves. Moreover, none of the patients for two pharmacists were interviewed in phase-2. This limited the exploration of a wider participant experience with regard to the feedback process between the different pharmacists.

#### Recommendations

In light of study findings, a distillation of key points should be considered to facilitate feedback collection in the future, these include the following:

- Use a third person.
- Follow a consistent approach.
- Collect patient feedback as soon as possible following pharmacists’ consultations.
- Maintain patient’s privacy when collecting feedback.
- Explain to patients that they can leave the competed questionnaire on the bed when discharged.
- Approach patients at a convenient time (e.g., allow enough time after surgery) for feedback collection.
- Collect patient feedback every 1–2 years for at least three months duration.

Future studies could investigate the implementation of the above-mentioned suggestions and its impact on pharmacists’ CSs. The use of electronic devices in collecting patient feedback was also suggested to facilitate obtaining results quickly, and to reduce the burdens associated with collecting completed questionnaires. This was not investigated by the current study and can be evaluated in the future. Additionally, involving carers who are acting as patients’ representatives in a consultation could represent a potential future application.

## Conclusions

The study provides valuable information to the field of patient feedback and pharmacy consultations. Findings support that collecting patient feedback on hospital pharmacists’ CSs is feasible. Participants viewed the feedback to be helpful, useful, and can play a role in enhancing pharmacists’ performance. All participants also expressed willingness to be engaged in such activity again. However, pharmacists need to be supported to help facilitate the process of feedback collection, especially within the context of hospital pharmacy setting. Recommendations were given to amend certain aspects of the process for the future, such as by assigning a third person to collect patient feedback. Most viewed the ISQ as a suitable tool with some suggestions to make it more relevant to hospital pharmacy.

## Supporting information

S1 Table Data collected by pharmacists’ participants (Phase-1). (DOCX)

S1 Appendix (DOCX)
