# Supplementary material for: Patient feedback on hospital pharmacists’ consultation skills: A feasibility study using the Interpersonal Skills Questionnaire (ISQ)
Source: PLoS One. 2022 Jul 14;17(7):e0268544. doi: 10.1371/journal.pone.0268544 (PMC9282534; doi:10.1371/journal.pone.0268544)
Supplement: S1 Table — (DOCX) [file pone.0268544.s001.docx]

**Data collected by pharmacists’ participants (Phase-1)**

| Pharmacist’s no. |  | Inpatient or outpatient | Patient’s gender | Age | 1^ST^ time to see pharm. | Patient said yes? | | Reason for not participating if given | Who recruited patient | Notes |
| --- | --- | --- | --- | --- | --- | --- | --- | --- | --- | --- |
| **Ph-A** | 1 | Inpatient | F | > 60 yo | Yes | Yes | - | | 3^rd^ person – another pharmacist |  |
|  | 2 | Inpatient | F | > 60 | Yes | Yes | - | | 3^rd^ person – another pharmacist |  |
|  | 3 | Inpatient | M | > 60 | Yes | Yes | - | | 3^rd^ person – another pharmacist |  |
|  | 4 | Inpatient | F | > 60 | Yes | Yes | - | | 3^rd^ person – another pharmacist |  |
|  | 5 | Inpatient | F | 25-59 | Yes | Yes | - | | Pharmacist |  |
|  | 6 | Inpatient | M | >60 | Yes | Yes | - | | Pharmacist |  |
|  | 7 | Inpatient | M | >60 | Yes | Yes | - | | Pharmacist |  |
|  | 8 | Inpatient | F | 25-59 | Yes | Yes | - | | 3^rd^ person – Pharmacy technician |  |
|  | 9 | Inpatient | M | 25-59 | Yes | Yes | - | | 3^rd^ person – Pharmacy technician |  |
|  | 10 | Inpatient | M | 25-59 | Yes | Yes | - | | 3^rd^ person – Pharmacy technician |  |
|  | 11 | Inpatient | F | > 60 | Yes | Yes | - | | 3^rd^ person – Pharmacy technician |  |
|  | 12 | Inpatient | M |  |  | Yes | - | | 3^rd^ person – Pharmacy technician | ISQ not returned |
|  | 13 | Inpatient | M |  |  | Yes | - | | 3^rd^ person – Pharmacy technician | ISQ not returned |
|  | 14 | Inpatient | M | > 60 | No | Yes | - | | 3^rd^ person – Pharmacy technician |  |
|  | 15 | Inpatient | M | > 60 | Yes | Yes | - | | 3^rd^ person – Pharmacy technician |  |
|  | 16 | Inpatient | M | > 60 | Yes | Yes | - | | 3^rd^ person – Pharmacy technician |  |
|  | 17 | Inpatient | M | > 60 | Yes | Yes | - | | 3^rd^ person – Pharmacy technician |  |
|  | 18 | Inpatient | M | 25-59 | Yes | Yes | - | | 3^rd^ person – Pharmacy technician |  |
|  | 19 | Inpatient | M |  |  | Yes | - | | 3^rd^ person – Pharmacy technician | ISQ not returned |
|  | 20 | Inpatient | F | >60 | NA | Yes | - | | 3^rd^ person – Pharmacy technician |  |
|  | 21 | Inpatient | F | >60 | Yes | Yes | - | | Pharmacist |  |
|  | 22 | Inpatient | F | >60 | Yes | Yes | - | | Pharmacist |  |
|  | 23 | Inpatient | M | >60 | Yes | Yes | - | | Pharmacist |  |
|  | 24 | Inpatient | M | > 60 | Yes | Yes | - | | Pharmacist |  |
|  | 25 | Inpatient | M | > 60 | Yes | Yes | - | | Pharmacist |  |
|  | 26 | Inpatient | F | > 60 | Yes | Yes | - | | Pharmacist |  |
|  | 27 | Inpatient | F |  |  | Yes | - | | Pharmacist | ISQ not returned |
|  | 28 | Inpatient | M |  |  | Yes | - | | Pharmacist | ISQ not returned |
|  | 29 | Inpatient | M |  |  | Yes | - | | Pharmacist | ISQ not returned |
|  | 30 | Inpatient | M | > 60 | Yes | Yes | - | | Pharmacist |  |
|  | 31 | Inpatient | M | > 60 | Yes | Yes | - | | Pharmacist |  |
|  | 32 | Inpatient | F | > 60 | Yes | Yes | - | | Pharmacist |  |
|  | 33 | Inpatient | M | > 60 | Yes | Yes | - | | Pharmacist |  |
|  | 34 | Inpatient | F | > 60 | Yes | Yes | - | | Pharmacist |  |
|  | 35 | Inpatient | F | 25-59 | Yes | Yes | - | | Pharmacist |  |
|  | 36 | Inpatient | F | 25-59 | Yes | Yes | - | | Pharmacist |  |
|  |  |  |  |  |  |  |  | |  |  |
| **Ph-B** | 1 | Inpatient | F | > 60 | Yes | Yes | - | | 3^rd^ person – Pre-reg pharmacist |  |
|  | 2 | Inpatient | M | 25-59 | Yes | Yes | - | | 3^rd^ person – another pharmacist |  |
|  | 3 | Inpatient | F | 25-59 | Yes | Yes | - | | Pharmacist |  |
|  | 4 | Inpatient | F | > 60 | Yes | Yes | - | | Pharmacist |  |
|  | 5 | Inpatient | F | 25-59 | Yes | Yes | - | | Pharmacist |  |
|  | 6 | Inpatient | F | 25-59 | Yes | Yes | - | | 3^rd^ person – Pre-reg pharmacist |  |
|  | 7 | Inpatient | M | > 60 | NA | Yes | - | | Pharmacist |  |
|  | 8 | Inpatient | M | > 60 | Yes | Yes | - | | Pharmacist |  |
|  | 9 | Inpatient | F | >60 | Yes | Yes | - | | Pharmacist |  |
|  | 10 | Inpatient | M | >60 | Yes | Yes | - | | Pharmacist |  |
|  |  |  |  |  |  |  |  | |  |  |
| **Ph-C** | 1 | Inpatient | M | 25-59 | No | Yes | - | | Pharmacist |  |
|  | 2 | Outpatient | F | 25-59 | Yes | Yes | - | | Pharmacist |  |
|  | 3 | Inpatient | M | 25-59 | Yes | Yes | - | | pharmacist |  |
|  | 4 | Outpatient | F | > 60 | Yes | Yes | - | | Pharmacist |  |
|  | 5 | Outpatient | M | > 60 | Yes | Yes | - | | Pharmacist |  |
|  | 6 | Outpatient | M | ? | ? | Yes | - | | pharmacist | ISQ not returned |
|  | 7 | Outpatient | F | >60 | Yes | Yes | - | | Pharmacist |  |
|  | 8 | Outpatient | F | >60 | NA | Yes | - | | Pharmacist |  |
|  | 9 | Outpatient | F | 25-59 | Yes | Yes | - | | Pharmacist |  |
|  |  |  |  |  |  |  |  | |  |  |
|  |  |  |  |  |  |  |  | |  |  |
| **Ph-D** | 1 | Outpatient | M | 25-59 | No | Yes | - | | 3^rd^ person – Dietitian |  |
|  | 2 | Outpatient | F | 25-59 | No | Yes | - | | 3^rd^ person – Dietitian |  |
|  | 3 | Outpatient | F | < 25 | No | Yes | - | | 3^rd^ person – Dietitian |  |
|  | 4 | Inpatient | F | > 60 | Yes | Yes | - | | 3^rd^ person – Pharmacy technician |  |
|  | 5 | Outpatient | M | 25-59 | No | Yes | - | | 3^rd^ person – Dietitian |  |
|  | 6 | Outpatient | F | <25 | No | Yes | - | | Pharmacist |  |
|  | 7 | Outpatient | M | 25-59 | No | Yes | - | | 3^rd^ person - Physiotherapist |  |
|  |  |  |  |  |  |  |  | |  |  |
| **Ph-E** | 1 | Inpatient | F | 25-59 | Yes | Yes | - | | Pharmacist |  |
|  | 2 | Inpatient | F | > 60 | Yes | Yes | - | | Pharmacist |  |
|  | 3 | Inpatient | F | - | - | Yes | - | | Pharmacist | Completed ISQ not returned |
|  | 4 | Inpatient | M | 25-59 | Yes | Yes | - | | 3^rd^ person – another pharmacist |  |
|  | 5 | Inpatient | F | 25-59 | Yes | Yes | - | | 3^rd^ person – another pharmacist |  |
|  | 6 | Inpatient | F | > 60 | Yes | Yes | - | | 3^rd^ person – another pharmacist |  |
|  | 7 | Inpatient | M | >60 | Yes | Yes | - | | Pharmacist |  |
|  | 8 | Inpatient | M | 25-59 | Yes | Yes | - | | Pharmacist |  |
|  | 9 | Inpatient | F | <25 | Yes | Yes | - | | Pharmacist |  |
|  | 10 | Inpatient | M | >60 | Yes | Yes | - | | Pharmacist |  |
|  | 11 | Inpatient | M | >60 | Yes | Yes | - | | Pharmacist |  |
|  | 12 | Inpatient | F | - | - | No | Patient says “would have done it but cannot see to write down answers” | | Pharmacist |  |
|  | 13 | Inpatient | F | - | - | No | Would do, but patient has difficulty with writing | | Pharmacist |  |
|  | 14 | Outpatient | F | 25-59?? | Yes | Yes | - | | Pharmacist |  |
|  | 15 | Inpatient | F | >60 | Yes | Yes | - | | Pharmacist |  |
|  | 16 | Outpatient | M | > 60 | Yes | Yes | - | | Pharmacist |  |
|  | 17 | Inpatient | F | > 60 | Yes | YES | - | | Pharmacist |  |
|  | 18 | Inpatient | M | > 60 | Yes | Yes | - | | Pharmacist |  |
|  | 19 | Outpatient | M | 25-59 | Yes | Yes | - | | Pharmacist |  |
|  | 20 | Outpatient | F | 25-59 | Yes | Yes | - | | Pharmacist |  |
|  | 21 | Outpatient | F | 25-59 | Yes | Yes | - | | Pharmacist |  |
|  | 22 | Inpatient | F | > 60 | Yes | Yes | - | | Pharmacist |  |
|  | 23 | Inpatient | F | 25-59 | Yes | Yes | - | | Pharmacist |  |
|  | 24 | Outpatient | M | - | - | No | Patient did not to wait | | Pharmacist |  |
|  | 25 | Outpatient | M | 25-59 | No | Yes | - | | Pharmacist |  |
|  | 26 | Outpatient | M | ? | ? | No | Did not want to wait | | Pharmacist |  |
|  | 27 | Inpatient | M | 25-59 | Yes | Yes | - | | Pharmacist |  |
|  | 28 | Inpatient | F | >60 | Yes | Yes | - | | Pharmacist |  |
|  | 29 | Inpatient | M | ? | ? | No | Did not want to wait, would have done it if the questionnaire could have been taken home to complete | | Pharmacist |  |
|  | 30 | Outpatient | F | 25-59 | Yes | Yes | - | | Pharmacist |  |
|  | 31 | Outpatient | M | ?not stated on ISQ | ? not stated on ISQ | Yes | - | | Pharmacist |  |
|  | 32 | Inpatient | M | >60 | Yes | Yes | - | | Pharmacist |  |
|  | 33 | Outpatient | F | 25-59 | Yes | Yes | - | | Pharmacist |  |
|  | 34 | Outpatient | M | 25-59 | Yes | Yes | - | | Pharmacist |  |
|  |  |  |  |  |  |  |  | |  |  |
| **Ph-F** | 1 | Outpatient | F | >60 | Yes | Yes | - | | 3^rd^ person – another pharmacist |  |
|  | 2 | Inpatient | M | 25-59 | No | Yes | - | | 3^rd^ person – another pharmacist |  |
|  | 3 | Inpatient | F | >60 | Yes | Yes | - | | 3^rd^ person – pharmacy technician |  |
|  | 4 | Inpatient | F | NA | NA | Yes | - | | 3^rd^ person – another pharmacist |  |
|  | 5 | Inpatient | F | - | - | No | Patient said that the pharmacist “just checked my pills and left, I don’t know him very well” | | 3^rd^ person – another pharmacist |  |
|  | 6 | Outpatient | M | <25 | Yes | Yes | - | | Pharmacist |  |
|  | 7 | Inpatient | F | 25-59 | Yes | Yes | - | | Pharmacist |  |
|  | 8 | Inpatient | F | >60 | Yes | Yes | - | | Pharmacist |  |
|  | 9 | Outpatient | M | >60 | Yes | Yes | - | | Pharmacist |  |
|  | 10 | Inpatient | F | >60 | Yes | Yes | - | | pharmacist |  |
|  | 11 | Inpatient | F | > 60 | Yes | Yes | - | | 3^rd^ person - Nurse |  |
|  | 12 | Outpatient | M | 25-59 | Yes | Yes | - | | Pharmacist |  |
|  | 13 | Outpatient | F | > 60 | Yes | Yes | - | | Pharmacist |  |
|  | 14 | Inpatient | F | >60 | Yes | Yes | - | | Pharmacist |  |
|  | 15 | Inpatient | F | 25-59 | Yes | Yes | - | | Pharmacist |  |
|  | 16 | Outpatient | F | > 60 | Yes | Yes | - | | Pharmacist |  |
|  | 17 | Outpatient | M | > 60 | Yes | Yes | - | | Pharmacist |  |
|  | 18 | Outpatient | F | 25-59 | Yes | Yes | - | | Pharmacist |  |
|  | 19 | Outpatient | M | 25-59 | Yes | Yes | - | | Pharmacist |  |
|  | 20 | Inpatient | M | > 60 | Yes | Yes | - | | Pharmacist |  |
|  | 21 | Inpatient | M | > 60 | Yes | Yes | - | | Pharmacist |  |
|  | 22 | Outpatient | M | 25-59 | Yes | Yes | - | | Pharmacist |  |
|  | 23 | Inpatient | F | 25-59 | Yes | Yes | - | | Pharmacist |  |
|  | 24 | Inpatient | M | > 60 | Yes | Yes | - | | Pharmacist |  |
|  | 25 | Outpatient | F | > 60 | Yes | Yes | - | | Pharmacist |  |
|  | 26 | Outpatient | F | 25-59 | Yes | Yes | - | | Pharmacist |  |
|  | 27 | Outpatient | M | NA | NA | Yes | - | | Pharmacist |  |
|  | 28 | Inpatient | M | >60 | Yes | Yes | - | | Pharmacist |  |
|  | 29 | Outpatient | F | > 60 | Yes | Yes | - | | Pharmacist |  |
